# On-Machine LTS Integration for Layer-Wise Surface Quality Characterization in MEX/P

**DOI:** 10.3390/s24113459

**Published:** 2024-05-27

**Authors:** Alejandro Fernández, Pablo Zapico, David Blanco, Fernando Peña, Natalia Beltrán, Sabino Mateos

**Affiliations:** Department of Construction and Manufacturing Engineering, University of Oviedo, 33203 Gijón, Asturias, Spain; afs@uniovi.es (A.F.); zapicopablo@uniovi.es (P.Z.); penafernando@uniovi.es (F.P.); nataliabeltran@uniovi.es (N.B.); sabino@uniovi.es (S.M.)

**Keywords:** laser triangulation, additive manufacturing, layer-wise scanning, MEX/P

## Abstract

Material Extrusion (MEX) currently stands as the most widespread Additive Manufacturing (AM) process, but part quality deficiencies remain a barrier to its generalized industrial adoption. Quality control in MEX is a complex task as extrusion performance impacts the consistency of mechanical properties and the surface finish, dimensional accuracy, and geometric precision of manufactured parts. Recognizing the need for early-stage process monitoring, this study explores the potential of integrating Laser Triangulation Sensors (LTS) into MEX/P manufacturing equipment for layer-wise 3D inspections. Using a double-bridge architecture, an LTS-based sub-micrometric inspection system operates independently from the manufacturing process, enabling comprehensive digitization and autonomous reconstruction of the target layer’s topography. Surface texture is then computed using standardized indicators and a new approach that provides insight into layer quality uniformity. A case study evaluating two alternative extruder head designs demonstrates the efficacy of this integrated approach for layer quality characterization. Implementing a generalized layer-wise procedure based on this integration can significantly mitigate quality issues in MEX manufacturing and optimize process parameter configurations for enhanced performance.

## 1. Introduction

Additive Manufacturing (AM) enables the fabrication of objects layer by layer instead of conventional manufacturing methods [1]. Analysis of current industrial trends forecasts sustained growth in the AM market over the next few years. Projections suggest that the market value will increase from USD 12.6 billion in 2020 to USD 44.5 billion in 2026 [2]. However, despite advancements, AM parts still fail to achieve manufacturing quality levels comparable to conventional alternatives [3]. Consequently, researchers underscore quality issues as the primary technological barrier hindering the wider industrial adoption of AM [3,4,5]. Intensive research has been conducted in recent years to mitigate this problem by detecting defects [6,7], enhancing part quality [8], and minimizing manufacturing errors [9,10].

Material extrusion of polymers (MEX/P) ranks among the most popular AM categories, largely owing to its affordability and user-friendly nature. Consequently, there is a sustained and ongoing research effort focused on enhancing the quality of MEX/P-produced parts. Particularly relevant are the efforts to apply process monitoring techniques in MEX/P, as reflected by Oleff et al. [11] and Fang et al. [12]. On-machine process monitoring could have three main non-excluded objectives: first, to minimize losses associated with processing defective parts through early detection of process incidents and deposition anomalies [13]; second, to ensure the manufacturing quality by comparing quantifiable aspects of the parts with reference values [14,15]; and third, to enable procedures for optimizing the quality of manufactured parts through closed-loop control routines [16].

Layer-wise or asynchronous monitoring implies gathering information on the part being manufactured layer-by-layer. In layer-wise monitoring, data are used to compute different quality metrics at each layer incrementally as the object is built up, allowing for asynchronous but on-process feedback and quality control. Layer-wise strategies can complement real-time (synchronous) monitoring, which provides immediate feedback during material deposition [17,18,19]. Nevertheless, conventional off-machine inspection of the final part typically carried out in metrological and mechanical characterization facilities, remains necessary to ensure comprehensive quality assessment [20]. Layer-wise strategies enable early detection of issues such as layer misalignment, warping, delamination, or other defects that may arise during the printing process. By monitoring each layer independently, manufacturers can promptly identify and address manufacturing issues, reducing the likelihood of producing faulty parts and minimizing material waste.

Although particularly popular in metal AM [21,22,23], layer-wise monitoring has also been applied in MEX/P, primarily through three different sensing technologies: thermal sensing [24], 2D vision [25,26], and 3D vision [16,23,27,28,29,30,31,32,33,34,35,36,37,38,39,40]. Monitoring the 3D layer topography offers distinct advantages over thermal and two-dimensional sensing, as it allows for direct measurement of layer features, like layer height [27,28], or in-plane defects [16,23,30,31]. Two technologies are mainly used for 3D monitoring of layer geometry: structured light photogrammetry [32,33,34,39] and laser triangulation (LT) [16,23,29,31,35,37,38,40]. Research endeavors employing structured light are challenged by the relatively low accuracy of such sensors. This constraint significantly shapes their utility, directing researchers to predominantly employ these devices for defect detection and qualification [32,33,39], often focusing on defects larger than 1 mm [39]. Furthermore, owing to the large dimensions of the necessary equipment, integrating structured light-based monitoring into MEX/P equipment is truly challenging, resulting in sensors typically being positioned at fixed external locations [32,33,34,39]. On the contrary, LT-based monitoring offers superior accuracy and resolution for 3D reconstruction. While LT sensors (LTS) may be somewhat large for integration into small, low-budget MEX/P equipment, they are sufficiently compact for being incorporated into medium and large manufacturing devices. Recently, there has been a growing research focus on LTS-based quality characterization for MEX/P parts. 

Thus, Faes et al. [27] built their own low-budget LTS with a USB microscope and then used it to determine height differences between tracks deposited with a syringe-based MEX machine as part of a closed-loop control effort. Lyu et al. [28], on the other side, located their LTS in an actuated superstructure above the MEX/P equipment. This led to the selection of a medium-distance stand-off sensor (200 mm) with good z-axis repeatability (1 µm). In this work, the information gathered by the LTS was used to optimize layer height accuracy and layer area uniformity. Digitized point clouds were utilized to compute the actual Z-coordinate for each layer, subsequently adjusting the z displacement for the following layer.

Furthermore, they transformed the 3D data into a depth colormap image and employed it to identify anomalies in the deposited surface concerning an assumedly normal one. Accordingly, they could act upon configuration parameters, like extrusion temperature and feed rate, to reduce over-extrusion and under-extrusion issues. In a later work by Xu et al. [37], this research group processes digitized point clouds to quantify part warpage, comparing the areal extension presenting significant vertical deviation with respect to the whole layer extent. Through the classification of warpage into four severity levels, they obtain a reference that is subsequently used to train a machine learning predictive model of warpage based on acoustic emission. This work illustrates how asynchronous information provided by the LTS can be utilized to train a real-time operating model. In later studies, the same group converted point clouds into colormaps [25,38] that are used to label small, discretized areas of the layer according to four levels: under-printed regions, over-printed regions, normal-printed regions, and empty areas. Based on this information, an optimization model was developed to adjust the print speed and feed rate in subsequent layers, thereby enhancing their topographic quality [38]. Using a different arrangement, Lin et al. [30] integrated an LTS directly in the manufacturing bridge of a multi-material MEX/P machine and used it to identify simulated underfill and overfill defects, although the poor vertical resolution (50 µm) of the sensor only allowed for coarse defect detection. Lafirenza [35] positioned the LTS on a platform fixed to the machine frame. Accordingly, relative movements between the part and the sensor rely solely on the manufacturing equipment. With their LTS boasting a resolution of 5 µm, the primary objective of this study was to characterize height deviations within the actual layer and the theoretical one. Additionally, they developed their own quality indicators purportedly capable of characterizing not only dimensional errors but also identifying defects that affect surface quality. A similar setup was employed by Moretti et al. [36], wherein a low-budget LTS was securely attached to the manufacturing equipment, enabling the displacement of the manufacturing tray to execute the scanning movement. Their approach aimed to address the limitations of pure 2D optical imaging monitoring for contour recognition. Due to the low vertical resolution of the LTS arrangement, the authors proposed a binarization approach to accurately identify material/void regions, foregoing a topographic characterization of the layer. With this method, they were able to compare the expected deposition pattern with the actual one in internal lattice structures through a pixel-wise strategy.

The use of a high-resolution LTS for monitoring surface quality in MEX/P has been proposed by Lischenko et al. [29]. While their sensor was not actually incorporated into the manufacturing machine, they explored the possibilities of using surface texture measurements to characterize layer quality. Nevertheless, these authors discouraged the direct use of standard Ra or Rz roughness indicators for quality characterization of defective samples and, instead, used allegedly more defect-sensitive power spectral density. Finally, other authors focused on isolated deposition tracks instead of areal layer characterization. Thus, digitizing using LTS was employed by Armstrong et al. to implement a trajectory correction strategy in their extrusion-based 3D bio-printing equipment [16]. 

The analysis of previous research efforts in LTS-based layer-wise monitoring of MEX/P part quality leads to the definition of several key aspects, like the integration strategy, the vertical repeatability, or the objective quality characteristic. The summary in Table 1 shows how most authors decided to integrate commercial LTS models [16,28,29,30,35,37,38], although there are also examples of built-in-house sensor arrangements [27,36]. The layer-wise inspection could take place using pure on-machine solutions, where the LTS has been fully integrated into the manufacturing equipment [16,27,30,31,36] or has been attached to actuated [28,37,38] or fixed [29,35] off-machine arrangements. The scanning strategy and the relative movement between part and LTS are conditioned by the type of integration used. The metrological characteristics of each LTS largely condition the type of quality characterization. Thus, solutions with low vertical resolution are applied to part height monitoring [27] or coarse defects [30,35]. LTS with intermediate performance are more versatile and have been used to evaluate the underfill and overfill issues [28,35,38], layer height deviations [34,35,37], warpage [37] or even the accuracy of the deposition trajectory [16]. On the other hand, the characterization of surface texture requires a high-resolution LTS and has been mostly beyond the scope of research efforts in this field, although its usefulness has been explored in off-machine arrangements [29].

As can be observed, most applications do not fully exploit the 3D reconstruction of layer texture because the selected LTS does not provide sufficient resolution for such a task. In these cases, point clouds are usually processed to derive two-dimensional information [28,35,36,38] or height differences [27,31,35]. These approaches commonly give birth to works addressing defect recognition, such as under-printed or over-printed regions, resulting in areal characterization of defect extensions [28,36]. These methodologies require substantial research efforts to define custom quality indicators and develop pattern recognition and classification software. This process involves a training phase to create a computational model capable of characterizing layer quality. The potential of surface texture analysis is usually neglected, and standard industrial quality indicators are seldom used or dismissed outright, allegedly due to poor correlation with the underlying phenomena behind defective surfaces [29].

In the present work, a new method for evaluating the quality of MEX/P parts utilizing on-machine LTS-captured 3D surface information is presented. An LTS with sub-micrometric resolution is integrated into an independent inspection bridge as part of a MEX/P test bench. The configuration parameters of the LTS are adjusted to get the best reconstruction quality, considering the use of PLA as the base material. A method for computing surface topography from the point cloud and calculating surface texture quality parameters according to the ISO 21920 standard series [41] is then proposed, alongside a strategy for retrieving significant information from the target layer. This methodology enables early detection of deposition issues, as it can be applied within the initial ‘solid’ layers of the MEX/P component. To test the usefulness of this approach, a case study has been conducted to compare the results obtained with two different extruder heads.

There are several novel aspects of this work. To the best of our knowledge, this is the first time an LTS with a sub-micrometric Z resolution has been fully integrated into an independent co-planar inspection bridge in MEX/P. Such an arrangement allows for an asynchronous layer-wise inspection of manufactured layers throughout the whole extension of the manufacturing tray without limiting or interfering with deposition operations. Another key aspect is that the proposed strategy for surface quality characterization relies solely on well-known roughness and waviness quantitative indicators and does not require downgrading the 3D information into 2D data or training complex computer models. Following industrial practice and surface texture standards, the method has been tailored to fit the periodic characteristics of layer deposition patterns while also providing statistical evidence of deposition uniformity. The proposed method offers advantages over two-dimensional surface quality characterization methods, as these can only detect coarse individual defects and cannot be used to quantify surface quality according to industrial standards. It also overcomes the resolution and integration limitations associated with 3D structured light systems. Furthermore, compared to other LTS-based applications, our method provides higher resolution, which is critical for topography reconstruction, and a minimally invasive integration, allowing it to work within the same guiding system used for the manufacturing subsystem. Finally, the statistical approach helps to provide a comprehensive and consistent analysis of the whole surface, avoiding the generalization of localized phenomena. These advantages make the approach presented in this work a significant step forward in developing instrumented equipment capable of early monitoring of MEX/P part quality during manufacturing.

This paper is organized as follows: Section 2 describes the integration of the LTS in the MEX/P test bench, along with the extrinsic calibration procedure. The strategy for layer quality characterization is presented in Section 3. Section 4 provides a case study comparing the performance of two alternative extruder head designs. Section 5 discusses the proposed approach’s results and suitability for AM quality characterization. Finally, Section 6 summarizes the conclusions of this work.

## 2. Integration of the LTS in a Test Bench

### 2.1. LTS Characteristics

A Gocator 2410 laser line profile sensor (LMI Technologies GmBH, Berlin, Germany) has been selected for this work. This LTS has metrological-degree characteristics, including a 2 M 405 nm laser that provides cleaner data and highly reliable results (0.2 µm repeatability along the Z axis) even on shiny surfaces. This sensor includes natively the option to optimize various internal parameters, such as frequency ranges and exposure, based on the texture and color of the material being used. This capability allows the sensor to adapt to a variety of materials, optimizing scanning accuracy and efficacy. Relevant characteristics of this LTS are provided in Table 2.

### 2.2. LTS Integration

The goal of integrating the LTS into MEX/P equipment is to allow for the full digitization of the target layers, regardless of their position in the workspace. However, some configurations in the consulted literature involve a reduction in effective scanning space due to the integration of the LTS into an existing manufacturing bridge. Other configurations involve external setups that may suffer from occlusion or limited inspection range. A double-bridge MEX/P test bench has been utilized to address these issues. As usual, the extruder head was mounted on the manufacturing bridge, but an additional inspection bridge was designed to accommodate various optical sensors. The mechanical design of the test bench features both bridges sharing common linear guides, ensuring that the XY movement of the extruder and the UV movement of the LTS occur along precisely parallel planes. Both the U and V axes feature micrometric linear encoders for accurate capture of positions e_u_ and e_v_. 

**Figure 1:**
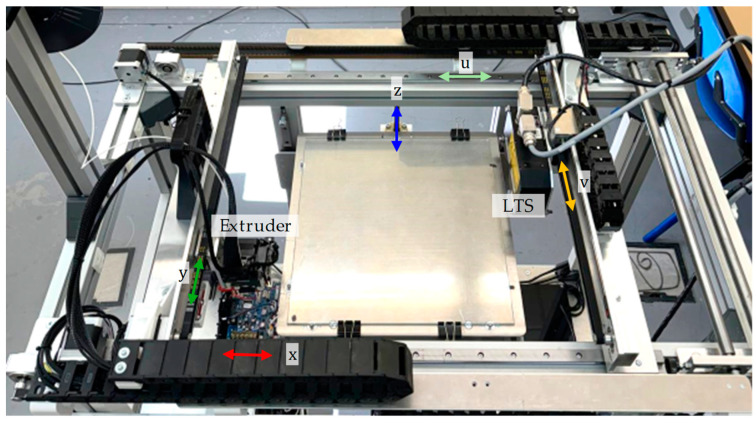
Double-bridge test bench with the extruder mounted on the manufacturing bridge and the LTS mounted on the inspection bridge.

The integration of the LTS into the test bench required certain modifications to the original design used in [42]. Therefore, a slight modification of the mechanical interface of the inspection bridge was carried out to achieve a robust holding of the LTS (Figure 1). 

In the first version of the test bench [42], the manufacturing bridge was controlled by an MKS Rumba board with an ATmega16U2 microcontroller, while control of the inspection bridge was performed through an Arduino Mega 2560. This approach hindered the communication and control tasks, while also being a non-optimal solution in terms of data handling. Accordingly, the design has been modified, and a single board, the Duet 3D Main Board 6HC (Duet3D Ltd., Cambridgeshire, UK), has been utilized to control the bench. This board can control up to six motors natively. It includes multiple inputs/outputs, Ethernet connectivity, and the option to incorporate additional expansion boards. It would also allow for closed-loop programming controlling additional axes, among other functionalities. By consolidating control onto a single board, communication and system control tasks have been greatly simplified. Additionally, the acquisition card for encoder reading used in [42] was suppressed, and a Zynq 7020 SoC (Xilinx, San Jose, CA, USA) was chosen to directly read the data. This change offers significant advantages, such as increased efficiency in data processing, lower power consumption, greater customization and flexibility, and the ability to implement more complex functions due to its integrated architecture. 

**Figure 2:**
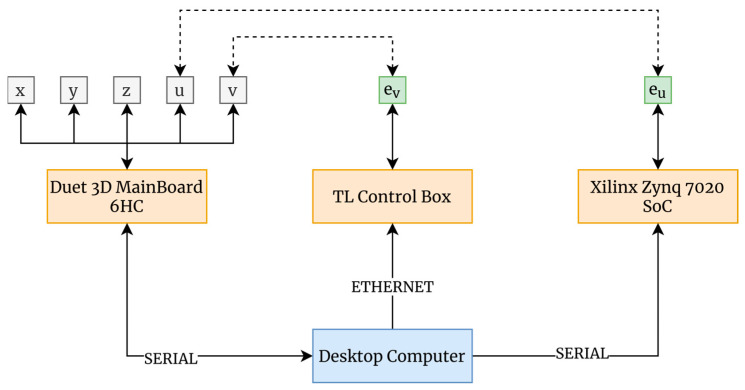
Test bench connection diagram. Dashed lines represent encoder monitoring, while solid lines represent physical connections.

In this case, a PC assumes the role of the master device, with a C++ application controlling the scanning system. This application will establish connections via serial ports to both the Duet3D and the Xilinx SoC. With these modifications, the connection diagram is shown in Figure 2.

### 2.3. Extrinsic Calibration

**Figure 3:**
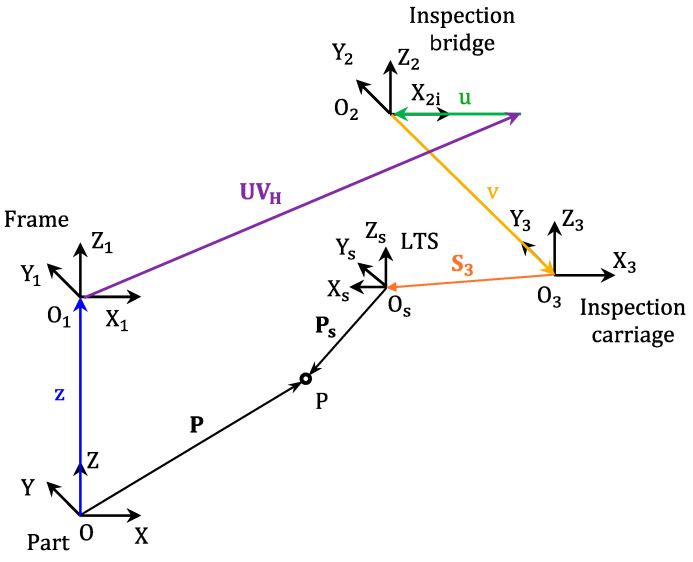
Kinematic chain for the extrinsic calibration of LTS.

The movements of the extruder during deposition operations are related to the workpiece reference system OXYZ. On the other hand, the information captured by the LTS is expressed according to a reference system $O_{S}X_{S}Y_{S}Z_{S}$, fixed to its own frame. In order to obtain all relevant information expressed according to the workpiece reference system, an extrinsic calibration of the LTS had to be performed. Accordingly, mathematical models based on the kinematics of the rigid solid [43] were used. These models define a reference system for each mechanical element on the kinematic chain that goes from a generic point on the workpiece, expressed in its reference system P, to that same point expressed in the reference system of the sensor $P_{S}$ (Figure 3).

Additionally, the movements of the machine axes involved are considered, as well as the possible offsets between them in the machine’s reference or home position (Figure 3). Regarding the mechanical part of the test bench, the kinematic model for extrinsic calibration considers the movement of the machine axes Z, U, and V (ZUV vector), as well as the offset existing between the machine home position of the XYZ axes (manufacturing) and the UV axes (inspection), ${UV}_{H}$. As for the LTS sensor, the kinematic model considers the offset between the reference system of this sensor and the carriage on which it is mounted, $S_{3}$, as well as a rotation matrix between these two systems, $R_{S}$. Thus, Equation (1) shows the extrinsic calibration model.

(1) $$P=ZUV+{UV}_{H}+S_{3}+R_{s}\cdot P_{s}$$

Therefore, the extrinsic calibration of the LTS will consist of determining the terms of vector $S_{3}$ and matrix $R_{S}$. For this matrix, because the LTS sensor supplies only two-dimensional data (specifically, $X_{S}$, and $Z_{S}$ coordinates), the problem is simplified to finding the six terms of the rotation matrix in its homogeneous form, as displayed in Equation (2).

(2) $$\left[\begin{array}{c} X_{P}\\ Y_{P}\\ Z_{P}\\ 1 \end{array}\right]=\left[\begin{array}{cccc} {R_{S}}_{11} & 0 & {R_{S}}_{13} & -u+{O_{UV}}_{x}+{S_{3}}_{x}\\ {R_{S}}_{21} & 0 & {R_{S}}_{23} & -v+{O_{UV}}_{y}+{S_{3}}_{y}\\ {R_{S}}_{31} & 0 & {R_{S}}_{33} & +z+{O_{UV}}_{z}+{S_{3}}_{z}\\ 0 & 0 & 0 & 1 \end{array}\right]\cdot \left[\begin{array}{c} X_{S}\\ 0\\ Z_{S}\\ 1 \end{array}\right]$$

**Figure 4:**
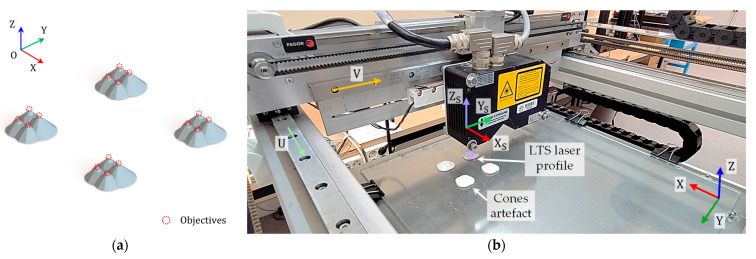
Digitization of an ad-hoc artifact for extrinsic calibration of the LTS: (**a**) 3D model of calibration cones; (**b**) Digitizing of the calibration artifact.

To determine the terms of the extrinsic calibration model, it was decided to use several pairs of conjugate points $P|P_{S}$. Each of these pairs represents the coordinates of a point expressed in both the workpiece reference system P, and the sensor reference system $P_{S}$, in addition to the values of the setpoints of the ZUV axes at the time of scanning of the respective point. To generate these pairs, similarly to other works [28,44,45], it was chosen to manufacture and digitize an ad-hoc artifact, in this case, composed of several cones of different heights (Figure 4a). These cones were digitized with the LTS recording the coordinates $P_{S}$ of 16 points, along with the corresponding positions of the ZUV axes at the time of scanning, i.e., z, u, and v, respectively. The coordinates in the workpiece system P were extracted from the CAD model of the artifact related to its manufacturing position in the XYZ axes (Figure 4b). 

Utilizing this data, the least squares method was applied to determine the unknown parameters of Equation (2), with the known offset $UV_{H}=[300,280,0]$. The results obtained are shown in Table 3. This model is reversible; given a desired position for a point to be digitized in the sensor system $P_{S}$, it is feasible to ascertain the machine setpoints z, u, and v required the ZUV axes to be moved accordingly.

## 3. Strategy for Quality Characterization of a Given Layer 

The strategy for quality characterization presented in this work is based on a statistical analysis of texture parameter variability throughout the entire extent of a solid layer. This approach takes advantage of the high Z-resolution provided by the LTS to extract an accurate 3D representation of layer topography with a high level of detail. Extracted point clouds are then processed to compute surface texture at multiple individual locations within the surface. Analyzing the variability of texture parameters, the proposed strategy can properly characterize the quality of the deposition. Such a method can only be used for solid layers. Nevertheless, MEX/P parts are usually arranged as a series of top and bottom solid layers with lightened layers in between. Accordingly, this strategy can be employed in the initial layers to acquire early feedback on processing issues and to adjust manufacturing parameters before investing additional time in a potentially defective part. 

### 3.1. Scanning Procedure

The scanning procedure is designed to extract a complete 3D digitization of the target layer. Since scanning is time-intensive, the procedure is optimized to minimize the time needed to capture a comprehensive 3D point cloud encompassing all the deposited material. 

**Figure 5:**
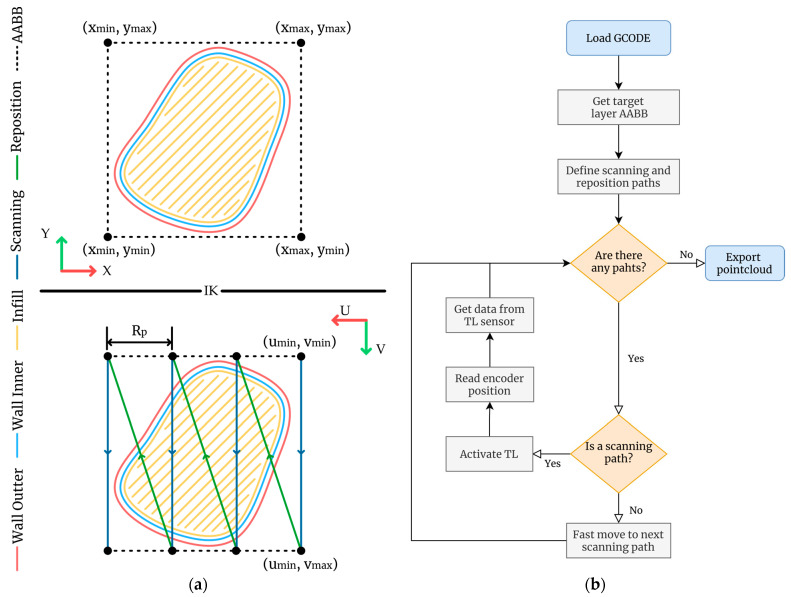
Scanning procedure: (**a**) definition of scanning and repositioning paths for a generic part. (**b**) flowchart for target layer data capture.

The process involves the following steps:

1. Determine target layer contour geometry and retrieve the correspondent information from the manufacturing file.
2. Extract the axis-aligned bounding box (AABB) that completely encloses contour geometry according to the workpiece reference system (Figure 5a).
3. Offset the AABB to ensure that the layer geometry is contained in the scanning area.
4. Apply the inverse kinematics (IK) described in Section 2.3 to the modified AABB and calculate the UV coordinates.
5. Calculate a scanning profile length (Rp) that is at least 10% shorter than the theoretical length.
6. Utilizing this Rp, along with the calculated UV coordinates, define the scanning and repositioning paths.

The outlined procedure has been implemented in a self-developed C++ application. Once the target layer has been selected, the software directly calculates the coordinates and sequence of scanning and repositioning paths. This information is then transmitted to the control board, line by line, through the serial port. Data capture occurs during the scanning trajectories and can be activated either via a timer or by utilizing Transistor–Transistor Logic (TTL) signals from the linear encoder. The data capture process employs the exposure and capture mode parameters optimized for each specific material, ensuring uniformity in terms of scanning conditions and spacing between different scanned profiles. Additionally, the position of the U-axis (e_u_) is continuously monitored by observing the signal from the linear encoder. Once the scanning process has been finished, a unique point cloud integrating the executed scanning trajectories represents the whole 3D topography of the target layer. Figure 5b provides a diagram of the described procedure.

### 3.2. Pre-Processing of Topographic Data

The obtained point cloud constitutes a representation of the target layer’s topography. However, it must undergo a pre-processing stage before being suitable for the calculation of surface texture parameters. Initially, the retrieved point cloud consists of a collection of Xs, Zs coordinates captured by the LTS inextricably related to the ZUV coordinates of the LTS simultaneously with each profile’s digitizing. This information must be converted into a collection of points with their relative positions expressed concerning the workpiece reference system XYZ. To achieve this, the extrinsic calibration described in Section 2.3 is applied. Once completed, the resultant point cloud must be processed to eliminate geometric information that is irrelevant to the characterization of surface texture and minimize noisy data. 

**Figure 6:**
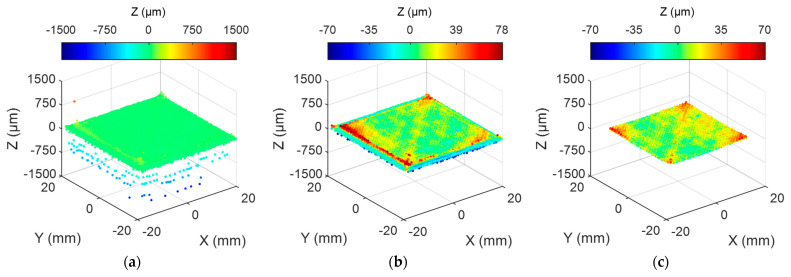
Different states of the point cloud during processing: (**a**) point cloud from extrinsic calibration; (**b**) point cloud of the target layer; (**c**) isolated target area for topography analysis.

First, points corresponding to layers behind the target layer shall be filtered out. The scanning procedure has already limited the extension of the point cloud to the approximate boundaries of the target layer. Although a reduced portion of points corresponding to lower layers can still be captured, most points effectively correspond to the target layer. Accordingly, an adjustment of the point cloud to a plane by means of the *singular value decomposition* method, followed by a calculation of the mode for the registered distance of captured points, provides a reference value for the whole target layer. A threshold of 75% of layer height ($L_{h}$) is thereafter used to extract the relevant information. Then, a new adjustment to a plane is performed, and outliers are filtered using a ±3σ threshold. A final step is applied to obtain the patch of interest within the digitized surface. Figure 6 contains an example of the pre-processing procedure tailored for the geometry of a square-shaped deposition layer.

### 3.3. Calculation of Surface Texture Parameters

Once the pre-processing step is completed, a linear interpolation model adjustment is performed, allowing the Z-coordinate of the surface’s topography to be obtained for any XY position within the target area. This step is required to retrieve a Z-coordinate value for intermediate positions between digitized points and makes use of the fit function (MATLAB, R2023b MathWorks, Carlsbad, CA, USA) to calculate the Z-position of a specified XY coordinate considering the corresponding values of neighboring points. This adjustment also allows for the extraction of surface profiles in a direction different from that of the scanning profile at the time of capture, a critical aspect for surface characterization, as explained below. 

Once the interpolation model has been obtained, the surface texture parameter can finally be calculated. Calculation of surface texture is covered by the 17.040.20 (Properties of Surfaces) ICS category as defined by the International Organization for Standardization (ISO) [46]. In the case of MEX/P parts, a surface with periodic marks is obtained, similar to what occurs with other manufacturing technologies, such as machining. This fact implies that the analysis of texture quality could be carried out using the profile method. The direction of test profiles should be perpendicular to the most prominent marks observed on the surface to fulfill the requirement of evaluating texture considering the highest possible roughness value. The ISO 16610 standard [47] establishes a series of Gaussian filters for the profile method that discriminate the primary surface profile, P, from the digitized surface profile or *skin model*. In a subsequent filtering stage, the waviness profile, W, and the roughness profile, R, will be extracted from the primary profile. The ISO 21920 standard [41] establishes the configuration of these filters based on the specified setting class. As the aim of the proposed strategy is to characterize the surface quality rather than to verify it according to a given specification, the setting class has been set to Sc4, based on the texture characteristics observed in preliminary tests. Selected setting class establishes a cut-off wavelength for the high-frequency filter, $\lambda_{s}$ of 8 µm and a cut-off wavelength for the low-frequency filter, $\lambda_{c}$, of 2.5 mm. Likewise, it establishes an evaluation length, $l_{e}$, of 12.5 mm composed of 5 sections of length, $l_{sc}$, of 2.5 mm each. This is the default filtering configuration defined for *mechanical profiles*. Nevertheless, a non-contact scanning sensor like the LTS demands a slight adjustment of this configuration to attenuate the intrinsic noise associated with optical sensors. Such a modification would not be required when using mechanical sensors due to the unavoidable morphological filtering of the probe tip radius.

Accordingly, $\lambda_{s}$ was modified using a ratio $\lambda_{c}/\lambda_{s}=30$ as described in [48]. This configuration does not alter the overall shape or magnitude of the profile and allows for the attenuation of the appearance of spurious points derived from the characteristic noise of the LTS. According to [41], the profile position shall be located on the part of the surface on which critical values can be expected, identified through a visual inspection. Therefore, this condition could hardly be automatized without an image-based qualitative clustering of the surface’s apparent quality. Nevertheless, this standard provides a valid alternative for quantitative characterization, since it states that if the location of the most critical part of the surface cannot be clearly identified, uniformly distributed traces shall be taken to represent the entire surface [41]. This method allows for the distribution of numerous traces across the target surface. As a result, a collection of surface profiles is obtained and processed to calculate the arithmetic average of profile height deviations from the mean line, as described in the ISO 21920 series. This parameter can be particularized for the waviness (Wa) and roughness (Ra) profiles using Equation (3).

(3) $$Wa,Ra=\frac{1}{l}\int_{L=0}^{L=l}\left|Z(L)\right|dL$$

where l is the evaluation length ($l_{W}$ for waviness and $l_{R}$ for roughness).

## 4. Case Study

A case study was conducted to demonstrate how the data from the integrated LTS can be used to assess the quality of manufactured parts. This test aimed to evaluate whether the texture information obtained through the proposed method correlates with changes in process conditions and/or machine design. The goal was to determine if this approach could be used to identify variations in part quality.

### 4.1. Test Configuration

**Figure 7:**
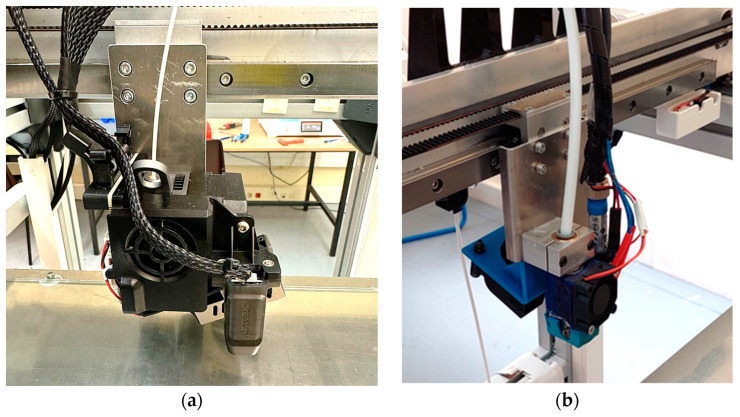
Extruder heads used in the case study: (**a**) commercial (E1); (**b**) proprietary (E2).

As stated in Section 2.2, the LTS has been integrated into a test bench used to evaluate alternative process configurations or to compare the performance of different components. The case study presented in this work compares the quality of parts manufactured with identical process parameters using two alternative components: a Sprite Extruder Pro Kit extruder head (Shenzhen Creality 3D Technology Co., Ltd., Shenzhen, China) (E1) and a proprietary extruder head (E2). Figure 7a contains an image of the commercial extruder head (E1), while an image of the proprietary extruder head (E2) is provided in Figure 7b. 

A square 40 × 40 mm^2^ layer was selected as the test target. Subsequently, 40 × 40 × 10 mm test specimens were designed and manufactured using both extruders. For test purposes, a common manufacturing configuration has been defined for both extruders. Table 4 contains the values for the main extrusion parameters. 

In the context of the proposed quality characterization strategy, texture analysis shall be performed on the bottom or top solid layers. In accordance with this, the last top layer was pointed out as the target layer. Hence, on-machine digitizing of the target layers on every test specimen was performed after they had been left to rest for a period of 30 min. This interval is crucial to warrant consistency in the digitization operations, ensuring accurate and reliable measurements, as it allows for the upper surface of the pieces to cool completely. Following the procedure described in Section 3, contours were suppressed, and a final target area of 30 × 30 mm^2^ was obtained. Traces were then homogeneously distributed, fixing a minimum distance of 0.8 mm between adjacent ones, and a total of 44 texture profiles were extracted from each sample. 

**Figure 8:**
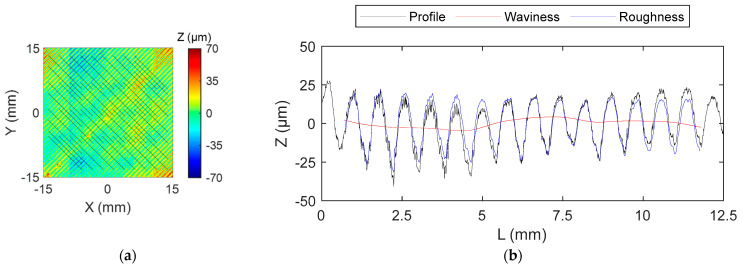
(**a**) Distribution of 44 pre-defined traces (black lines) over the target area (15 × 15 mm^2^) overlaid upon a colormap representation of the Z-coordinates from a sample specimen; (**b**) example of superimposed profiles corresponding to one single trace (primary, roughness and waviness).

Figure 8a shows the distribution of profiles across the target area, while an example of an extracted profile alongside the superimposition of the correspondent waviness and roughness profiles is presented in Figure 8b.

### 4.2. Results

**Figure 9:**
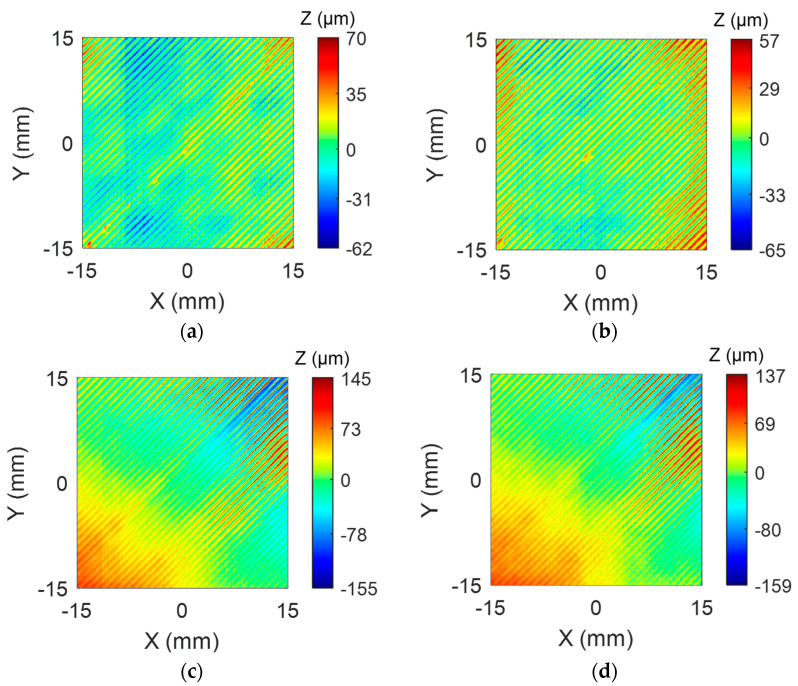
Surface digitizing of test specimens: (**a**) E1—first replicate; (**b**) E1—second replicate; (**c**) E2—first replicate; (**d**) E2—second replicate.

Tests were carried out in accordance with the experimental plan, and two replicates of each specimen were manufactured, with their corresponding target layers digitized. Extracted point clouds were processed following the procedure defined in Section 3.1. Figure 9 contains colormaps representations of the Z-coordinates for layers manufactured with both E1 and E2 extruder heads. 

At first sight, surface quality appears to be quite different for both extruder heads. Although not entirely uniform, topographic differences among E1-manufactured specimens are lower than those observed in E2 specimens. Considering the range of Z values, E1 samples show height differences within the respective ranges 122 µm and 132 µm, whereas these ranges increase to 296 µm and 300 µm in the case of E2 specimens. Ra and Wa were selected as representative texture quality parameters for comparison purposes within the experimental plan. Therefore, Ra and Wa values were calculated from each individual profile for every single experiment following Equation (3). A statistical analysis of data distribution has shown that neither Ra nor Wa fits a normal distribution. Instead, utilizing the *individual distribution identification* tool provided by Minitab (Minitab, LLC., State College, PA, USA), it has been found that both texture parameters fit a three-parameter Weibull distribution. Accordingly, each set of data has been fitted and the distribution parameters—shape (β), scale (η) and location (γ)—were calculated. The shape parameter accounts for the skewness of the distribution and determines whether the distribution is symmetric, skewed to the right or skewed to the left. The scale parameter affects the spread or variability of the distribution. Finally, the location parameter specifies the position of the distribution along the x-axis. Other descriptive parameters include the mean ($\bar{T}$) and the standard deviation ($\sigma_{T}$), which can be calculated through Equations (4) and (5), respectively.

(4) $$\bar{T}=\gamma +\eta \cdot \Gamma \left(\frac{1}{\beta }+1\right)$$

(5) $$\sigma_{T}=\eta \cdot \sqrt{\Gamma \left(\frac{2}{\beta }+1\right)-\Gamma {\left(\frac{1}{\beta }+1\right)}^{2}}$$

**Figure 10:**
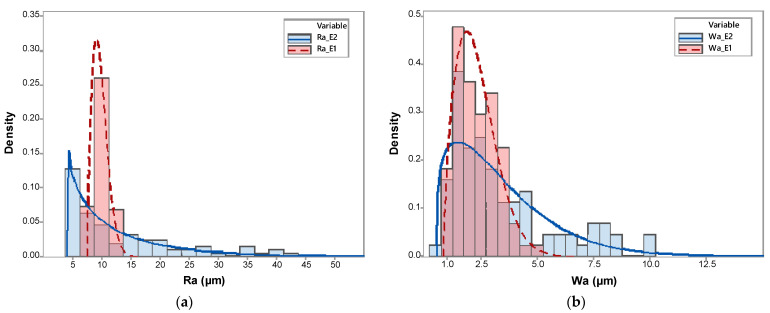
(**a**) Histogram of Ra values for E1 and E2; (**b**) histogram of Wa values for E1 and E2.

Histograms of Ra and Wa distributions are provided in Figure 10. 

Statistics calculated for each set of data have been summarized in Table 5. Results support the differences observed in Figure 9 since clear differences have been quantified. Concerning Ra, the shape parameter for the E1 specimens indicates that the probability distribution is slightly positively skewed and shows a right tail, whereas, for the E2 specimens, the distribution decreases monotonically beyond the value of the location (Figure 10a). The larger scale for E2 indicates that the distribution stretches, which can also be observed through the higher standard deviation values.

Overall, E2 specimens show higher mean values and a much higher variability for Ra than E1 parts. This indicates that the E2 extruder head produces surfaces with a worse quality in terms of roughness. Regarding Wa, a similar situation has been found, although in this case, both distributions show positive shape values and right tales can be observed in corresponding histograms (Figure 10b). Wa distribution for E2 specimens is stretched with respect to E1; mean values are higher while standard deviation is much higher, indicating that the E2 extruder head also produces surfaces with worse waviness.

## 5. Discussion

Quality improvement in MEX/P could be achieved through various strategies, but it will undoubtedly require reliable data describing both the production process and the resulting outcome. Monitoring layer quality stands out as a promising strategy since, contingent upon the selected instrumentation, data collection could span from layer geometry to temperature maps.

Integrating the LTS into the manufacturing machine is not an easy achievement. Some authors have chosen to develop their own low-cost systems [27,36], but the resulting resolution significantly limits their applicability to topographic reconstruction. The size and weight of the sensors often necessitate mounting the LTS on a superstructure independent of the manufacturing system, with few examples of integration on the inspection bridge [16]. Our proposal to integrate the LTS into an inspection subsystem implemented through an independent bridge that shares guiding elements offers clear advantages. Firstly, it prevents overloading the manufacturing bridge with the additional weight of the LTS, thus maintaining standard manufacturing conditions without affecting inertia. Secondly, it allows operation on the same displacement plane used for manufacturing, avoiding parallelism issues and simplifying calibration and coordinate conversion between the manufacturing and inspection subsystems. Thirdly, it enables the LTS to be placed close to the layer to be inspected, allowing the use of high-resolution LTS with low clearance distance. Thus, there is no need to position the LTS at a considerable distance from the layer to avoid interference with the extrusion system [28,37,38,40], thereby avoiding the need to compromise on high-resolution LTS. The mechanical arrangement of the integration, as discussed, enables the use of an LTS with sub-micrometer resolution and excellent repeatability characteristics. This is crucial for achieving a sufficiently high-quality reconstruction of the surface topography of the layer, which can be subsequently used to characterize surface topography through standardized roughness or waviness parameters. This characteristic would be difficult to achieve with other integration approaches, which explains why almost all the reviewed studies focused on the identification of defects like warping or over-extrusion [28,30,35,37,38,40] instead of addressing texture characterization. In fact, the characterization of quality through surface texture would be limited to systems like the one proposed here, mounting an LTS with high resolution and repeatability. The proposed system’s capability to offer a thorough scan of an individual layer enables a characterization strategy grounded in the statistical analysis of the distribution of texture parameter values obtained from multiple measurements within the target area. According to the proposed strategy, the point cloud will be processed to extract multiple individual profiles following the guidelines outlined in well-established industrial standards [41].

Consequently, roughness and waviness parameters are calculated separately for each profile. The overall analysis is then centered on examining the variability of these parameters across the entire surface area. Manufacturing through MEX/P does not exhibit the stability characteristics typical of machining processes such as turning or milling. Therefore, the process variability and, associated with it, the variability of the surface texture of the manufactured layers serve as suitable criteria for characterizing the quality of the manufacturing process or, as in the proposed case study, comparing the results obtained under different manufacturing conditions.

The case study conducted in this work illustrates the potential of processing the three-dimensional information about layer surface collected using an LTS to evaluate the effects upon deposition quality of different components, like the extruders used here. The comparison between the surface quality provided by a commercial extruder E1 and that achieved with the proprietary one E2 has allowed the identification and characterization of anomalies. Results for E2-manufactured surfaces present worse texture characteristics than those manufactured with the commercial extruder head. This phenomenon can be ascertained through both Ra and Wa parameters. Overall, the E2 extruder provides higher Ra and Wa values while, at the same time, the variability of results within different profiles is more significant than that calculated for E1 specimens. Since the selected manufacturing configuration was the same throughout all the experiments, this result points out that differences in the design or mechanical disposition of the E2 extruder head have an impact on the stability and, consequently, the quality of material deposition. In this case, the research team has concluded that this difference is related to an inappropriate location of the layer fan with respect to the nozzle. Regardless of the specific reasons behind the observed differences, the key factor presented in this work is using an integrated surface reconstruction system combined with effective data processing. This approach allows for precise characterization of performance differences using quantifiable data.

Despite its potential as a source of information about the ongoing process, integrating LTS into production machines has certain drawbacks, such as the high cost of accurate sensors and space requirements. Although low-cost LTS can offer useful information [27], their performance is lacking when it comes to detailed 3D reconstructions, such as those required for surface quality characterization. Additionally, accurate sensors are relatively bulky and heavy, which could be a problem for most commercial MEX/P equipment. A test bench like the one described in this paper has been designed to provide enough room for the integration of bulky sensors, which is not the usual situation in commercial equipment. Precise but expensive LTS should be limited to high-end equipment and industrial production, where the potential of layer-wise LTS-based topographic reconstruction aligns with stringent quality specifications. Each company would need to assess which models within their equipment range are suitable for the approach presented in this work. Beyond these limitations, the results obtained in this study confirm the utility of introducing an inspection bridge equipped with an LTS into test benches to evaluate the quality of parts manufactured using newly designed components, optimized deposition strategies, or alternative configurations of process parameters.

The integration of an LTS into MEX/P equipment opens the door to developing multiple studies on the influence of a wide variety of factors on the quality of the deposited layers. These studies could span from process configuration parameters to component design or the dynamics of material deposition. The example provided in this paper can provide insight into the potential uses of a layer-wise inspection approach. When integrated into test benches, LTS could provide relevant information regarding the development and fine-tuning of technological variants. Future works could further delve into characterizing the relationship between layer topography and extrusion parameters (flow, temperature, speeds, etc.). The influence of the feedstock on the quality of digitization with LTS is another aspect to analyze, to determine its potential significance in materials with different optical properties (color, transparency, etc.). Additionally, specific research could be conducted on the relationship between layer topography and the mechanical properties of the parts. On a different note, the integration of a layer-by-layer inspection subsystem into commercial equipment poses significant challenges, as previously mentioned. The viability of this integration will be contingent upon the specific market orientation of the equipment. However, triangulation-based scanning technology is not the sole option that could enable 3D reconstruction of layer topography. Still, it is the one that exhibits more favorable characteristics for industrial applications in terms of accuracy and robustness. In this context, one of the prospective lines of research would be to assess the capability of alternative scanning technologies to deliver results comparable to those obtained in this study while concurrently overcoming some of the mentioned limitations. Particularly, it would be ideal to explore more cost-effective technologies that could be easier to incorporate. Advancements in this direction would facilitate the expansion of the layer-wise inspection strategy to machines targeting more economical segments and, ultimately, promote its widespread adoption in the MEX/P domain or even in other AM processes.

## 6. Conclusions

Achieving quality improvement in MEX/P processes requires access to reliable data regarding both the production process and its outcome. Hence, monitoring layer quality emerges as a promising strategy. This study explores the potential of integrating an LTS into MEX/P manufacturing equipment for layer-wise 3D inspection and is focused on the reconstruction of the target layer’s topography for surface quality characterization. Integrating an LTS into the manufacturing equipment poses significant challenges that had been addressed by using an independent bridge. This arrangement offers numerous advantages, such as optimal weight distribution, precise operational alignment, and close proximity to the inspected layer, which facilitates the use of high-resolution LTS without compromising quality. The proposed system enables thorough scanning of individual layers, facilitating a characterization strategy grounded in statistical analysis of texture parameters. A complete digitization of the target area allows for the extraction of multiple uniformly distributed traces, providing a robust characterization of roughness and waviness parameters distribution and variability along the entire surface. This detailed approach ensures a comprehensive understanding of the surface quality, which is critical for improving the overall manufacturing process. The case study presented in this paper confirms the utility of integrating an inspection bridge equipped with LTS into test benches for evaluating part quality and adjustment of process configuration. This integration shall help researchers study the influence of various factors on layer quality, from process parameters to component design. Surface quality differences between a commercial extruder and a proprietary extruder has been characterized, and the results showed that the proprietary one generates surfaces with worse texture characteristics, evidenced by higher Ra and Wa and greater variability. This circumstance is related to the mechanical design or the proprietary extruder, specifically the disposition of the layer fan. Accordingly, it has been probed that the surface texture of solid layers reflects the variability of material deposition operations, serving as a suitable criterion for characterizing how different manufacturing conditions affect the quality of the part. The potential use of the proposed approach spans from exploring the relationship between layer topography, extrusion parameters, and mechanical properties to evaluating alternative scanning technologies for 3D reconstruction. On-machine layer-wise inspection strategies could, therefore, become a key element in increasing the quality of MEX/P manufactured parts. This approach contributes to reducing the gap with conventional manufacturing processes in terms of reliability and enhances the potential for high-quality, consistent production in MEX/P technologies.

## Figures and Tables

**Table 1:** Summary of publications on LTS-based layer-wise MEX/P monitoring.

Ref.	Sensor Type	Integration	Repeatability	Quality Characteristic
[16]	Keyence LJ-G030	On-Machine Attached to the manufacturing bridge	1 µm	Deposition trajectory
[27]	Built in-house	On-Machine Attached to the manufacturing bridge	N/A Error > 100 µm	Part height
[28]	Keyence LJ-V7200	Off-Machine Independent structure with linear actuators	1 µm	Under-extrusion Over-extrusion
[29]	Keyence LJ-820	Off-Machine Fixed to a superstructure	0.3 µm	Surface texture
[30]	Micro-Epsilon scanControl 2600-50	On-Machine Attached to the manufacturing bridge	N/A Resolution = 50 µm	Severe underfill Severe overfill
[31]	N/A	On-Machine Attached to the extruder head	N/A Resolution = 2 µm	Layer height
[35]	Micro-Epsilon scanControl 2900-50 BL	Off-Machine Fixed to a superstructure	N/A Resolution = 4 µm	Layer height Defective surface
[36]	Built in-house	On-Machine Fixed to the structure	N/A	Layer contour
[37]	Keyence LJ-V7200	Off-Machine Independent structure with linear actuators	1 µm	Warpage
[38,40]	Keyence LJ-V7200	Off-Machine Independent structure with linear actuators	1 µm	Under-extrusion Over-extrusion

**Table 2:** Gocator 2410 technical specification.

Characteristic	Value
Dimensions (L × W × H) (mm)	44 × 99 × 145
Scan Rate (Hz)	200–5000
Measurement Range (MR) (mm)	6.0
Profile Length ^1^ (mm)	9.92–10.60
Data Points/Profile (points)	1710
Profile Resolution (μm/point)	5.8–6.2
Clearance distance (mm)	19.0
Repeatability ^2^ (μm)	0.2
Linearity ^2^ (±% of MR)	0.015
Laser Class	2M
Encoder/Trigger input	TTL

^1^ Dependent on sensor-to-profile distance. ^2^ Measured in the sensor-to-profile direction.

**Table 3:** Parameters of the kinematic calibration model obtained.

Element	Description	Component	Units	Value
$S_{3}$	Offset between LTS and inspection carriage	${S_{3}}_{X}$	mm	48.830
		${S_{3}}_{Y}$		−18.492
		${S_{3}}_{Z}$		−0.398
$R_{S}$	Rotation matrix of LTS	${R_{S}}_{11}$	-	−1.0396
		${R_{S}}_{21}$		0.0128
		${R_{S}}_{31}$		−0.0041
		${R_{S}}_{13}$		−0.0009
		${R_{S}}_{23}$		−0.0264
		${R_{S}}_{33}$		0.9885

**Table 4:** Main parameters of the extrusion process with the extruders.

Parameter	E1	E2
Layer height (mm)	0.2	0.2
Flow rate (%)	100	100
Filament diameter (mm)	1.75	2.85
Top/bottom pattern	Lines	Lines
Wall ordering	Outside–inwards	Outside–inwards

**Table 5:** Statistic indicators of Ra and Wa distributions calculated from test specimens.

Extruder Head	Texture Parameter	β	η	γ	$\bar{T}$ (µm)	$\sigma_{T}$
Ra	E1	1.769	2.513	7.656	9.9	1.3
	E2	0.879	8.441	4.274	13.3	10.3
Wa	E1	1.747	1.689	0.854	2.4	0.9
	E2	1.285	3.074	0.564	3.4	2.2

## Data Availability

The authors will make the raw data on which the conclusions of this article are based available upon request.
